# Detecting a hierarchical genetic population structure: the case study of the Fire Salamander (*Salamandra salamandra*) in Northern Italy

**DOI:** 10.1002/ece3.1335

**Published:** 2015-01-16

**Authors:** Giulia Pisa, Valerio Orioli, Giulia Spilotros, Elena Fabbri, Ettore Randi, Luciano Bani

**Affiliations:** 1 Department of Environmental and Earth Sciences, University of Milano-BicoccaPiazza della Scienza 1, Milano, I-20126, Italy; 2 Department of Biology, University of Milanovia Celoria 26, Milano, I-20133, Italy; 3 Laboratory of Genetics, Istituto Superiore per la Protezione e la Ricerca Ambientale (ISPRA)Ozzano Emilia, BO, I-40064, Italy

**Keywords:** Amphibians, broad-leaved forests, fragmented population, microsatellites, STRUCTURE software

## Abstract

The multistep method here applied in studying the genetic structure of a low dispersal and philopatric species, such as the Fire Salamander *Salamandra salamandra*, was proved to be effective in identifying the hierarchical structure of populations living in broad-leaved forest ecosystems in Northern Italy. In this study, 477 salamander larvae, collected in 28 sampling populations (SPs) in the Prealpine and in the foothill areas of Northern Italy, were genotyped at 16 specie-specific microsatellites. SPs showed a significant overall genetic variation (Global *F*_ST_ = 0.032, *P *< 0.001). The genetic population structure was assessed by using STRUCTURE 2.3.4. We found two main genetic groups, one represented by SPs inhabiting the Prealpine belt, which maintain connections with those of the Eastern foothill lowland (PEF), and a second group with the SPs of the Western foothill lowland (WF). The two groups were significantly distinct with a Global *F*_ST_ of 0.010 (*P *< 0.001). While the first group showed a moderate structure, with only one divergent SP (Global *F*_ST_ = 0.006, *P *< 0.001), the second group proved more structured being divided in four clusters (Global *F*_ST_ = 0.017, *P *= 0.058). This genetic population structure should be due to the large conurbations and main roads that separate the WF group from the Prealpine belt and the Eastern foothill lowland. The adopted methods allowed the analysis of the genetic population structure of Fire Salamander from wide to local scale, identifying different degrees of genetic divergence of their populations derived from forest fragmentation induced by urban and infrastructure sprawl.

## Introduction

Habitat destruction and degradation are considered the most important threats for wild populations, but the effects produced by the overall habitat loss are often complex to understand because many impacting factors do not act separately, but rather they play cumulatively or interactively (Giplin and Soulé 1986; Lindenmayer 1995; Young et al. 1996). These factors can shape the landscape, triggering a fragmentation process that affects the spatial distribution of animal populations by confining them to residual habitat fragments with a consequent reduction of population size. Small populations are usually more vulnerable to intrinsic demographic and genetic threatening factors, such as a higher variance of birth and death rates which leads to a higher probability of extinction and a less effective demographic response to environmental stochasticity. Moreover, small populations suffer from a higher genetic drift and inbreeding, leading to a loss of heterozygosity and genetic variability (Keller and Waller 2002; Frankham 2003, 2005). The mating of closely related individuals causes the inbreeding depression that has negative effects on demography (e.g., juvenile fitness and mortality rate among offspring; see Ralls et al. 1988; Lacy 1993; Lacy and Lindenmayer 1995), reducing population growth rates and thus the population size. In addition, the decrease of genetic diversity makes populations less adaptable to environmental variability (Frankham 2005). The smaller the population is, the more important are the effects of intrinsic threatening factors (Giplin and Soulé 1986).

Fragmentation processes generate metapopulations that are defined as a network of spatially discrete populations (i.e., subpopulations) linked by dispersal (Hanski and Simberloff 1997). The amount of dispersal between subpopulations represents the degree of their ecological connectivity. Several species naturally live in metapopulations, but their long-term persistence could be threatened by anthropogenic habitat fragmentation. Indeed, this process could lead to isolation, which is the disruption of dispersal movements and, consequently, the halting of the gene flow between subpopulations, which further emphasizes the negative effects produced by the habitat loss. Knowledge of the ecology of fragmented populations is essential in order to prevent their isolation or even restoring the ecological connectivity between them (Saunders et al. 1987; Burgman and Lindenmayer 1998). We studied the Fire Salamander *Salamandra salamandra* (*AMPHIBIA*, *URODELA*) genetic population structure because amphibians are usually model candidates for studies of fragmentation effects on connectivity (Moore et al. 2011). As they generally have low dispersal capabilities (Caldonazzi et al. 2007; Allentoft and O’Brien 2010) and are rather philopatric to breeding sites (Blaustein et al. 1994), amphibians are particularly susceptible to isolation. These characteristics often determine a high genetic differentiation among populations, even at restricted spatial scales (Allentoft and O’Brien 2010).

In the study area, located in Northern Italy, the species distribution still covers a wide area, although some subpopulations have declined or become locally extinct, where anthropic pressure is strong. Here, broad-leaved forest ecosystems which represent its habitat are mainly threatened by degradation, reduction, and fragmentation. Thus, the species ecology and distribution look particularly suitable to perform a general screening of forest ecosystems connectivity.

Molecular markers, such as polymorphic proteins or DNA sequences, are widely used for evaluating the effective genetic connectivity between populations, because they can distinguish breeding events, measure migration rates between generations and estimate gene flow (Avise 1994; Frankham et al. 2002; Frankham 2006). Moreover, molecular techniques require a lower sampling effort, as they usually rely on biological samples collected in a single period (Neville et al. 2006).

We chose microsatellites as molecular markers, because they are widely used in genetic population structure analyses and they have already been identified for the Fire Salamander in discrete numbers (Steinfartz et al. 2004; Hendrix et al. 2010). Microsatellites pertain to a noncoding DNA part of the genome, with no known function. This “neutral” region of DNA is thus particularly useful as it could change over time without bias induced by selection pressures. However, we stress that microsatellites have sometimes been suspected to be not completely neutral, meaning that at least some of the variation observed within and among populations may be attributed to selection (Kauer et al. 2003). Microsatellite markers generally have high mutation rates resulting in high standing allelic diversity (Selkoe and Toonen 2006). For this reason, when used for evaluating the ecological connection between populations, they should be identified in sequences with mutation rates which are low relative to the migration rates of individuals (Beebee and Rowe 2004).

The traditional Bayesian approach for the evaluation of genetic population structure from microsatellite data consists in the assignment of a genotyped representative sample of individuals to genetically homogenous groups. Usually, the sample is analyzed in one stage, and results can only highlight the main genetic structure of studied populations at wide scale. Nevertheless, some studies have stressed the usefulness of a multistep approach (Vähä et al. 2007; Harris et al. 2014), which entail a separate re-analysis of the groups identified in the previous steps, in order to identify hierarchical and more detailed genetic structure (Evanno et al. 2005; Pritchard et al. 2010). We adopted this method, as it can be very useful in large study areas with potentially fragmented habitats where local highly divergent populations can be hidden by the overall wide-scale genetic population structure.

## Methods

### Study area and sampling design

**Figure 1:**
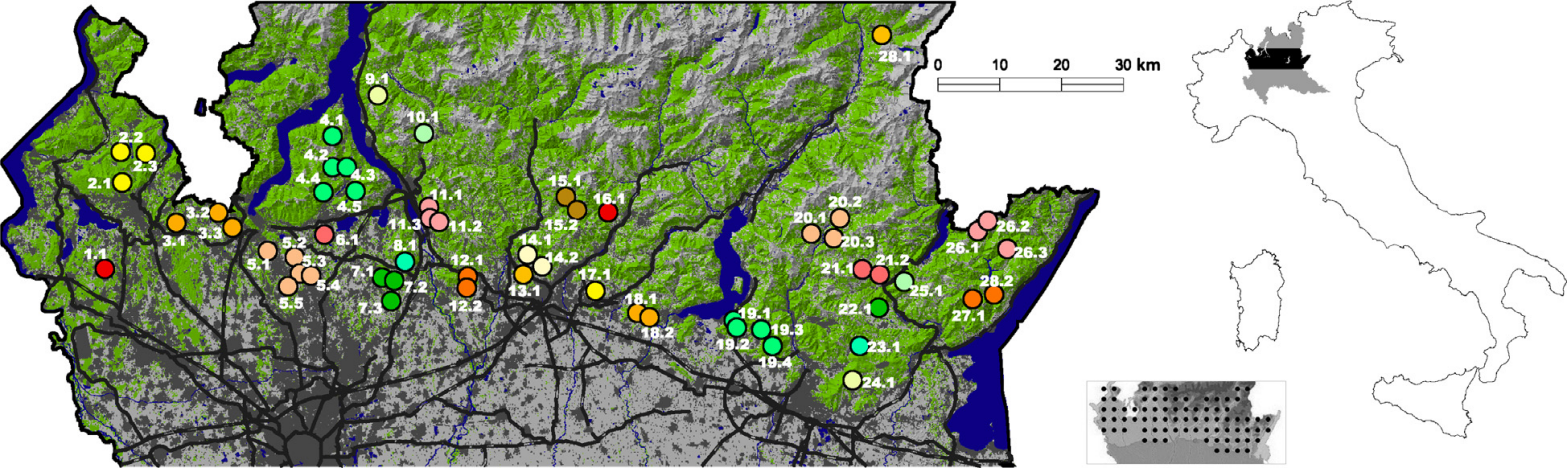
Study area colored according to land uses (left) and its geographical position within Italy (right): on the right, study area (black), within Lombardy (gray) in Northern Italy. On the left, forests in green, urban areas in dark–gray, farmland and other open habitats in gray, lakes, wetlands, and rivers in blue. Black lines represent main roads. Different colored dots represent sampling populations (SPs), while numbers refer to sampling locations (LOCPRIOR) with the first number indicating the SP which the sampling location pertain to. In the lower inset map, dots on the elevation map of the study area represent the Prealpine distribution of the Fire Salamander in Lombardy (the darker is the background, the higher is the elevation; from Di Cerbo and Razzetti 2004).

The study area is located in the Prealpine belt and in the foothill lowland of the Lombardy region (Northern Italy, Fig. 1). These areas were originally covered by extensive broad-leaved forests, which have been progressively removed and fragmented, especially during the last century. Forests have been replaced by a conspicuous urban sprawl particularly in the foothill lowland, while in the Prealps, they appear to be more continuous (Fig. 1).

**Figure 2:**
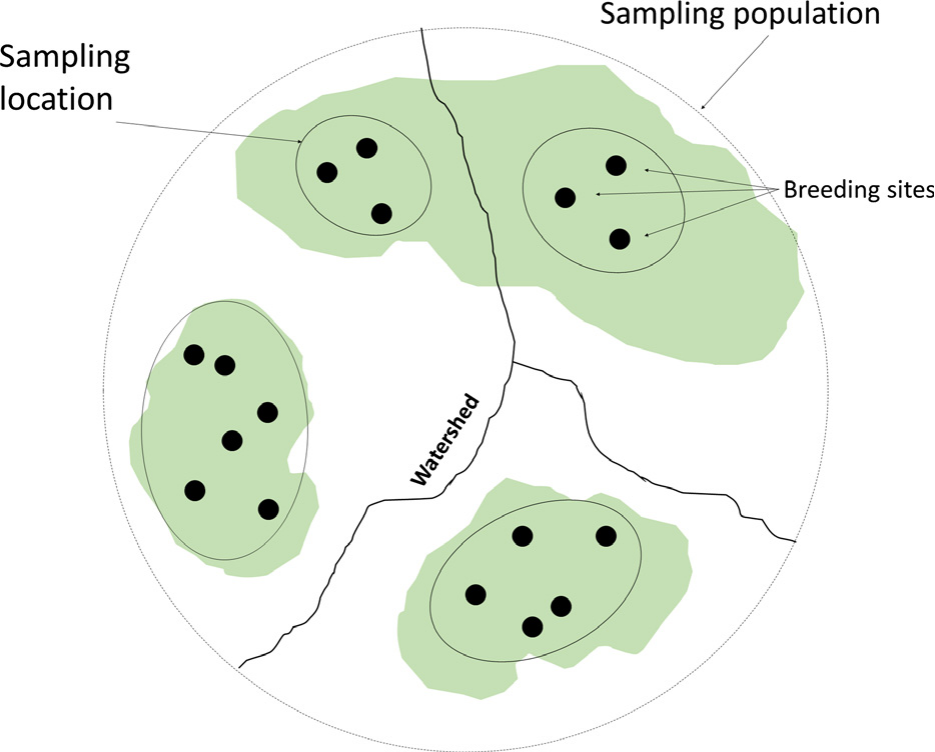
Sampling design of Fire Salamander larvae. Each sampling population (dotted circle) can contain a variable number of sampling locations (continuous-line ovals), which group all breeding sites (black dots) in the same continuous forest patch (green areas) and in the same watershed (black lines).

Sampling design was realized in order to collect genetic data over most of the species range in the study area. We identified 28 sampling populations (SPs; Fig. 1), inhabiting suitable areas separated from each other by anthropogenic (i.e., continuous urban areas, main roads, etc.) or natural barriers (i.e., lakes, main rivers) (min distance: 2900 m). Within each SP, we selected between 1 and 5 sampling locations (making a total of 57), according to Fire Salamander ecology, breeding sites availability, and area accessibility (distance between sampling locations within SPs ranges from 1300 m to 9700 m). In each sampling location, we identified between 1 and 15 breeding sites (making a total of 174) corresponding mainly to slow-flowing streams and their meanders (ponds, in some cases) located in forest areas. The sampling location contained all close breeding sites within the same continuous forest patch in the same watershed (the distance between breeding sites within the sampling locations ranges from 12 m to 4100 m) (Fig. 2). This sampling design was realized in order to reduce the probability of having only siblings within the same sample unit (sampling location).

We collected 477 tissue samples by cutting the tip (about 3–4 mm) of 1–4 salamander larvae tail in each breeding site, all year round, from 2010 to 2013. We chose not to sample more than four individuals per site, in order to avoid possible bias deriving from full-sibling individuals when inferring population genetic structure by sampling larvae in breeding sites (Goldberg and Waits 2010). Tissue samples were stored in 95% ethanol in the field and subsequently kept in the laboratory at −20°C.

Salamander larvae were captured and handled with permission of the Lombardy regional administration: P. T1.2009.0016990 decreed on 2009/09/16 by the Environment, Parks and Protected Areas General Directorate of the Lombardy Region (D.G. Ambiente, Parchi e Aree Protette della Regione Lombardia) for 2010–2012, and administrative order 964 decreed on 2013/02/11 by the Agriculture General Directorate of the Lombardy Region (D.G. Agricoltura della Regione Lombardia) for 2013–2014.

### DNA extraction and analyses of microsatellite markers

DNA was extracted using the Quick-g DNA™ MiniPrep kit (Zymo Research, USA) according to the manufacturer’s instructions, eluted in 180 *μ*L of elution buffer (10 mmol/L Tris-HCl, pH 8; 0.1 mmol/L EDTA), and stored at −20°C until subsequent handlings.

All samples were genotyped by polymerase chain reaction (PCR) for 20 species-specific microsatellite markers (Steinfartz et al. 2004; Hendrix et al. 2010).

PCR amplifications were carried out in a 10-*μ*L mix reaction containing: 1-*μ*L genomic DNA solutions from tissue extraction, 1 *μ*L of 10× PCR buffer with 2.5 mmol/L Mg^2+^, 2 *μ*L of bovine serum albumin (2%), 0.4 *μ*L of dNTPs 10 *μ*mol/L, and 0.2 or 0.3 *μ*L of primer mix (forward and reverse) 10 *μ*mol/L plus 0.25 units of Taq polymerase (5 PRIME Inc., Gaithersburg, USA) and purified water. PCR conditions were optimized for each primer pair, amplifications were performed in a 9700 ABI thermal cycler using the following protocol: (94°C × 2 min’), a number of cycles ranged from 30 to 40 at (94°C × 30 s’’) (annealing temperature × 30 s’’) (72°C × 30 s’’), and a final extension at 72°C for 10 min’. Some primer pairs (SalE8, SalE12, SalE14, SST-A6-II, and SST-B11) were amplified using a touchdown PCR starting from Ta+8°C to Ta for 8 cycles.

PCR products were analyzed in an Applied Biosystems 3130XL DNA sequencer (Life Technology, Inc.), and allele sizes were estimated using the software GENEMAPPER 4.0 (Life Technology, Inc.). Positive (known genotypes) and negative (no DNA) controls were used to check for laboratory contaminations, which never occurred. A 10% randomly selected subset of the other samples was PCR-replicated two times to check for allelic dropout and false alleles. Four of 20 microsatellite loci (Sal29, SST-F10, SST-G6, and SST-G9) were excluded from the analysis because we were not able to obtain PCR products that could be clearly interpreted.

We used Colony 2.0 (Jones and Wang 2010) to identify possible full-siblings, in order to avoid allele frequencies bias due to sampling larvae (Allendorf and Phelps 1981). We randomly selected one individual from each full-siblings dyad (Goldberg and Waits 2010) and removed the others from all further analyses. We used GenAlEx v. 6.501 (Peakall and Smouse 2006, 2012) to calculate the probability of identity (that is the probability of two independent samples having by chance the same identical genotype) in order to check whether the number of loci was suitable to identify univocally the individuals. The program LOSITAN was used to test for loci out of neutrality, which can influence the estimation of most population genetic parameters (Antao et al. 2008). The measures of the degree to which alleles at two loci were associated (linkage disequilibrium) was calculated using GENEPOP (Rousset 2008). We estimated the degree of genetic diversity among the 28 SPs, calculating global and pairwise *F*_ST_ (Wright 1931; Weir and Cockerham 1984), and we tested their significance by a randomization test based on 9999 permutations. These estimated probabilities were corrected using the sequentially rejective Bonferroni method (Holm 1979), that is more powerful than regular Bonferroni correction (Allendorf et al. 2013). Finally, we calculated population genetic parameters, such as the number of genotyped individuals (N), number of different alleles (Na), number of effective alleles (Ne), allelic range (AR), observed (Ho) and expected heterozygosity (He), and fixation index (*F*_IS_), for each locus on all individuals. Before performing genetic population structure analyses, we tested each locus for Hardy–Weinberg Equilibrium (HWE), checking its *F*_IS_, in order to detect an excess of homozygosity, which can bias population structure analyses. All these analyses were performed using GenAlEx v. 6.501. Nevertheless, as suggested by Allendorf et al. (2013), a deficit of heterozygosity may be caused by the presence of multiple demes (the Wahund effect, typically occurring in separate populations), sex linkage (occurring when some alleles are linked to one of the two sexes), and null alleles, but only the latter implies the removal of loci out of the HWE. We checked all loci for null alleles using Micro-Checker (Van Oosterhout et al. 2004) which can identify genotyping errors that can cause deviations from Hardy–Weinberg equilibrium and may bias population genetic analyses (Chapuis and Estoup 2007). Finally, we discharged only those loci not at equilibrium after sequentially rejective Bonferroni correction (see Allendorf et al. 2013), which also showed a probability to have null alleles.

### Fire Salamander genetic population structure

Genetic population structure was performed analyzing the biparental multilocus genotypes by means of the Bayesian clustering procedure implemented in STRUCTURE 2.3.4 (Pritchard et al. 2000; Falush et al. 2003, 2007; Hubisz et al. 2009), which was designed to identify genetically distinct clusters (populations of origin, K) of the sampled individuals. This analysis gave an assignment probability of each individual (*Q*_i_) pertaining to the identified populations of origin. Populations were constructed by minimizing the departures from HWE and linkage equilibrium, which could result from recent admixtures, migration, or hybridization.

When genetic data have a weak signal, the analysis of genetic population structure by means of a Bayesian approach (as the one used by this software) can be improved with the knowledge of the sampling location of each individual. In fact, including the sampling location as a prior in the model, the clustering algorithm assumes that the probability of assignment of each individual to a population of origin varies among locations, without finding population structure where it does not exist (Hubisz et al. 2009). For this reason, we used the sampling location as LOCPRIOR in all performed analyses (see Tucker et al. 2012; Walsh et al. 2012; Kovach et al. 2013; Parsons et al. 2013).

As we did not know whether studied populations were discrete or had an admixed ancestry, we chose to run STRUCTURE using two ancestry models: “no-admixture” and “admixture”. Actually, in some cases, it was unlikely that individuals from different SPs shared recent common ancestors (Falush et al. 2003), as the distances between SPs were higher than dispersal distance for several orders of magnitude. For this reason, we performed the analysis using the “no-admixture” model, which is also recognized to be more powerful in detecting subtle population structure (Pritchard et al. 2010). Nevertheless, in other cases, SPs could be relatively close and probably not completely isolated by barriers and might actually share an admixed ancestry. In these cases, the “admixture” model seemed to be more appropriate (Porras-Hurtado et al. 2013).

The effectiveness of the identification of populations of origin (K) may be further affected by the choice of the allele frequency model. Allele frequencies may show marked differences between distinct populations of origin and ancestral relationships between them is not expected (Rosenberg et al. 2005; Porras-Hurtado et al. 2013). In this case, the independent allele frequencies model seems to be more powerful in identifying populations highly divergent from each other (Pritchard et al. 2010) and reduces the likelihood of overestimating K (Hale et al. 2013). On the other hand, populations may show similar allele frequencies due to a shared recent ancestry (Pritchard et al. 2000; Rosenberg et al. 2005), and in this case, the correlated allele frequency model proves more powerful in detecting distinct populations of origin (Falush et al. 2003; Rosenberg et al. 2005; Porras-Hurtado et al. 2013).

The possible presence of different types of relationships between our SPs can hide a hierarchical structure that cannot be detected by a traditional one-step analysis. Indeed, in the presence of highly divergent populations, their separate analysis may improve the effectiveness of the correlated allele frequencies model in finding substructures (Evanno et al. 2005; Pritchard et al. 2010). For this reason, we adopted a multistep approach that consists of running STRUCTURE a first time in order to identify a main structure and then re-running the software separately for clusters identified in the previous step. Individuals were assigned to a cluster according to the best assignment probability (Evanno et al. 2005) of the sampling location (see LOCPRIOR above) in which they were collected. The process can be repeated until substructures of divergent populations are detected (see Vähä et al. 2007). At each step, we genetically characterized the identified clusters, testing all loci for HWE, and calculating *F*_IS_ for each of them. Thus, we removed all loci out of equilibrium after sequential Bonferroni correction.

At each step, we ran STRUCTURE according to four parameter combinations using the two possible settings for “ancestry models” and “allele frequency models”, because we did not have any information on the origins and the relationships of the sampled populations. All the runs for the “no-admixture” models were performed using a *burnin* period of 20,000 followed by 200,000 MCMC (Hastings 1970; Green 1995), while we chose to increment *burnin* period to 50,000 followed by 500,000 MCMC for the “admixture” models in order to reach an equilibrium in the estimated values of their key statistical parameters (see Porras-Hurtado et al. 2013). All the analyses were replicated 20 times for K ranging from 1 to 10. The optimal K values were selected by means of STRUCTURE HARVESTER (Earl and vonHoldt 2012) following the Evanno method (Evanno et al. 2005), based on the second order rate of change in the log probability of data between successive K values. In order to identify more detailed or subtle population structures, we also considered the K values with the highest average likelihood examining the plateau of the ln Pr(X|K) (Pritchard et al. 2000). The average population assignment probability *Q*_p_ and the average individual assignment probability *Q*_i_ were calculated using CLUMPP 1.1.2 with the Greedy algorithm (Jakobsson and Rosenberg 2007). The graphical displays (histograms) of genetic population structures were performed using the software DISTRUCT 1.1 (Rosenberg 2004), which plots individuals as colored bars according to their average *Q*_i_s.

Finally, we estimated the main genetic parameters of the groups identified in subsequent steps and calculated pairwise genetic distances among them in order to quantify the strength of genetic divergences and evaluate the ability of the multistep approach in discerning these genetic groups.

## Results

### Genetic variability

Among the 477 sampled individuals, the Colony software identified 26 full-sibling dyads, corresponding to 23 pairs and three triplets of full-siblings. After the removal of full-siblings, the whole dataset consisted of 448 individuals. All loci resulted neutral to selection from LOSITAN analysis after Holm–Bonferroni correction. The probability of identity for increasing locus combinations (16 loci) resulted in values as low as 7.8 × 10^−12^. Values lower than 0.01 are believed to adequate for population studies (Waits et al. 2001). The panel of microsatellites thus supported reliable individual genotype identification. All loci were polymorphic, with the number of different alleles (Na) ranging from 5 to 24, and the effective number of alleles (Ne) varying between 1.03 and 5.23. All these parameters, with allelic range (AR), observed (Ho) and expected (He) heterozygosity, and fixation index (*F*_IS_), for each locus, are shown in Table 1. We calculated *F*_ST_ statistics on a subsample (436 individuals) to allow convergence in GeneAlex algorithm, deleting locus Sal23 (which had a high number of no data) from the initial pool of 16 loci and four SPs (SP1, SP10, SP16, and SP28) with fewer than five individuals. Significant overall genetic variation was observed (15 loci: global *F*_ST_ = 0.032, *P *< 0.001). Pairwise *F*_ST_ between the 24 SPs ranged from 0.010 to 0.101, with 66 significant comparisons after Holm–Bonferroni correction. Sampling populations SP5, SP6, SP7, and SP12 showed the highest number of significant pairwise tests (Table 2). In the linkage disequilibrium test, no association between alleles at two loci within SPs was found. Two loci of 16 resulted not at HWE after Bonferroni correction and were removed from the further genetic population structure analyses (SalE12 and SSTB11; Table 1).

**Table 1:** Population genetic parameters for 448 salamander larvae collected in Lombardy (Northern Italy): number of genotyped individuals (*N*), number of different alleles (Na), number of effective alleles (Ne), allelic range (AR), observed (Ho) and expected heterozygosity (He), fixation index (*F*_IS_). Hardy–Weinberg Equilibrium test: degree of freedom (Df), chi-squared value (Chi-sq), *P*-value (*P*), significance after Holm–Bonferroni correction (HB sig).

Locus	Population genetic parameters							HWE test			
	*N*	Na	Ne	AR	Ho	He	*F*_IS_	Df	Chi-sq	*P*	HB sig
All loci	446.06 (1.28)	9.94 (1.26)	2.79 (0.29)	–	0.546 (0.042)	0.579 (0.047)	0.048 (0.013)	32	Inf.	≪0.001	sig
SalE2	448	11	2.32	216–262	0.560	0.569	0.015	55	34.41	0.987	ns
Sal3	448	11	2.49	181–327	0.569	0.598	0.048	55	54.26	0.503	ns
SalE5	448	5	1.03	180–190	0.027	0.027	−0.011	10	0.08	1.000	ns
SalE6	448	5	2.34	277–297	0.527	0.572	0.079	10	6.23	0.795	ns
SalE7	448	11	2.75	184–232	0.643	0.636	−0.010	55	33.04	0.992	ns
SalE8	446	10	4.26	143–181	0.731	0.765	0.045	45	64.92	0.027	ns
SalE11	448	6	2.21	238–258	0.513	0.548	0.064	15	12.95	0.606	ns
SalE12	441	16	4.42	223–307	0.621	0.774	0.197	120	740.25	≪0.001	sig
SalE14	448	6	2.19	237–257	0.527	0.543	0.030	15	5.86	0.982	ns
Sal23	428	9	2.46	282–320	0.549	0.594	0.076	36	45.60	0.131	ns
SST-A6-I	448	6	1.75	207–231	0.420	0.428	0.020	15	5.05	0.992	ns
SST-A6-II	448	8	2.34	193–221	0.578	0.573	−0.010	28	16.75	0.953	ns
SST-B11	447	24	5.19	149–263	0.763	0.807	0.055	276	434.29	≪0.001	sig
SST-C2	447	13	3.06	194–246	0.620	0.673	0.079	78	105.81	0.020	ns
SST-C3	448	5	1.67	207–227	0.400	0.400	0.001	10	5.72	0.838	ns
SST-E11	448	13	4.13	233–311	0.690	0.758	0.090	78	96.21	0.079	ns

sig, significant; ns, not significant.

**Table 2:** Pairwise *F*_ST_ among the 24 of 28 sampling populations (SPs). Bold values were statistically significant after a randomizations test (9999 permutations) with Holm–Bonferroni correction.

SP	SP2	SP3	SP4	SP5	SP6	SP7	SP8	SP9	SP11	SP12	SP13	SP14	SP15	SP17	SP18	SP19	SP20	SP21	SP22	SP23	SP24	SP25	SP26
SP2	–																						
SP3	0.013	–																					
SP4	0.017	0.021	–																				
SP5	**0.026**	**0.022**	**0.038**	–																			
SP6	0.040	0.039	0.040	0.036	–																		
SP7	0.022	**0.019**	**0.025**	**0.015**	**0.046**	–																	
SP8	0.041	0.023	0.048	0.027	0.060	0.022	–																
SP9	0.057	0.046	0.050	0.062	0.081	0.054	0.064	–															
SP11	0.015	0.018	0.013	**0.035**	0.048	**0.023**	0.036	0.038	–														
SP12	**0.025**	**0.019**	**0.029**	**0.020**	**0.048**	**0.021**	0.023	0.054	**0.025**	–													
SP13	0.042	0.040	0.035	0.059	0.062	0.045	0.066	0.055	0.029	0.047	–												
SP14	0.023	0.019	0.025	**0.038**	**0.051**	**0.026**	0.038	0.026	0.013	**0.030**	0.024	–											
SP15	0.049	0.048	0.054	**0.075**	0.064	**0.065**	0.066	0.061	0.046	**0.064**	0.073	0.048	–										
SP17	**0.054**	**0.039**	0.046	**0.049**	0.058	**0.039**	0.043	0.044	0.031	**0.044**	0.045	0.030	0.060	–									
SP18	0.034	0.032	0.038	**0.042**	**0.057**	**0.037**	0.035	0.045	0.024	**0.035**	0.041	0.024	0.067	0.028	–								
SP19	**0.037**	**0.031**	**0.043**	**0.034**	**0.064**	**0.028**	0.031	0.043	0.025	**0.032**	0.039	0.020	**0.075**	0.031	0.022	–							
SP20	**0.030**	0.025	**0.036**	**0.031**	**0.054**	**0.031**	0.039	0.033	0.023	**0.037**	0.042	0.018	0.054	**0.044**	0.033	0.028	–						
SP21	0.026	0.026	0.039	**0.038**	**0.057**	**0.037**	0.052	0.038	0.029	**0.038**	0.038	0.021	0.050	0.040	0.034	0.021	0.023	–					
SP22	0.023	0.021	0.028	**0.044**	0.049	**0.036**	0.043	0.040	0.016	**0.039**	0.027	0.010	0.043	0.035	0.024	0.029	0.022	0.023	–				
SP23	0.029	0.024	0.040	0.039	0.055	0.034	0.034	0.055	0.031	**0.039**	0.045	0.019	0.055	**0.050**	0.030	0.034	0.026	0.032	0.016	–			
SP24	0.030	0.029	0.037	**0.036**	**0.059**	0.026	0.034	0.052	0.024	0.021	0.035	0.020	0.063	0.040	0.033	0.018	0.034	0.032	0.031	0.037	–		
SP25	0.030	0.020	0.036	0.042	0.049	0.035	0.046	0.054	0.034	0.044	0.035	0.023	0.061	0.050	0.035	0.036	0.031	0.026	0.018	0.028	0.042	–	
SP26	0.033	0.029	0.041	**0.049**	0.057	**0.042**	0.050	0.042	0.027	**0.049**	0.040	0.017	0.060	0.040	0.025	0.029	0.028	0.027	0.012	0.027	0.040	0.021	–
SP27	**0.078**	0.060	0.066	**0.078**	0.092	**0.060**	0.070	0.076	0.063	**0.061**	0.057	0.043	0.101	**0.061**	0.063	0.044	0.064	0.057	0.054	0.064	0.053	0.047	0.046

### Fire Salamander population structure

**Figure 3:**
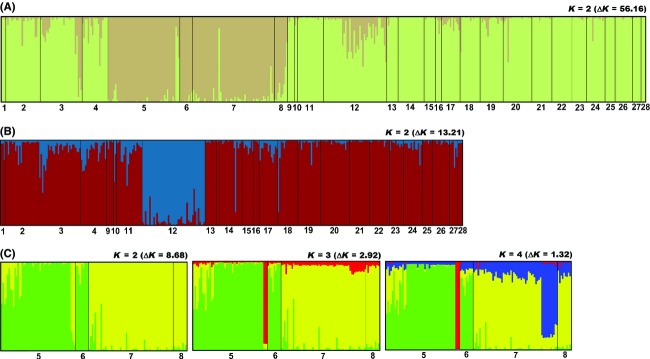
Inferred populations structures for (A) the whole sample (448 individuals, 28 sampling populations, SPs) that was divided in the PEF group (light green) and the Western foothill lowland (WF) group (light brown); (B) STRUCTURE analysis performed only on the PEF group (323 individuals, 24 SPs) showed the presence of two clusters corresponding to SP12 (blue) and all the others SPs (dark red); (C) the three histograms of the WF group (125 individuals, four SPs) showed population structures with 2, 3, and 4 clusters, respectively (see Results). For *K* = 4, cluster 1 in green, cluster 2 in red, cluster 3 in yellow, and cluster 4 in blue. These colors correspond to those in Fig. 4. Each bar represents an individual and is colored (using DISTRUCT) according to CLUMPP individual probability of assignment (*Q*_i_) obtained from “No-admixture” models with “correlated allele frequencies” and LOCPRIOR information.

The first step of population structure analysis, performed on all samples (28 SPs, 448 individuals), produced consistent results among the four parameter combinations. According to the Evanno method, we identified two distinct clusters (*K* = 2; see Fig. 3A for the “no-admixture” model after CLUMPP analysis). CLUMPP *Q*_p_ values, estimated by the four models, were always higher than 80% for both cluster 1 and 2, except for only one sampling population (SP12) for which the two “independent allele frequencies” models produced a *Q*_p_ value of 58% for cluster 1. For the same SP, the two “correlated allele frequencies” models estimated a *Q*_p_ value of 82% pertaining to cluster 1. For this reason, we considered the SP12 pertaining to cluster 1. The r LOCPRIOR parameter was always less than one, indicating that prior groups were informative in detecting population structure (Hubisz et al. 2009; Pritchard et al. 2010). The first cluster included all the Prealps area and the Eastern foothill lowland (and was named “PEF” group), with 24 SPs (323 individuals), while the second one corresponded to the Western foothill lowland (named “WF” group) with 4 SPs (from SP5 to SP8, 125 individuals; see Fig. 3A for the “no-admixture” model after CLUMPP analysis). Global *F*_ST_ for the whole sample divided in the two groups was 0.010 (14 loci: *P *< 0.001). See Table 3 for all population genetic parameters and the HWE test.

**Table 3:** Population genetic parameters for the PEF (323 individuals) and Western foothill lowland (WF) (125 individuals) groups: number of genotyped individuals (*N*), number of different alleles (Na), number of effective alleles (Ne), allelic range (AR), observed (Ho) and expected heterozygosity (He), and fixation index (*F*_IS_). Hardy–Weinberg Equilibrium test: degree of freedom (Df), chi-squared value (Chi-sq), *P*-value (p), significance after Holm–Bonferroni correction (HB sig).

		Population genetic parameters						HWE test			
Group	Locus	*N*	Na	Ne	Ho	He	*F*_IS_	DF	Chi-sq	*P*	HB sig
PEF	All loci	321.5 (1.43)	7.86 (0.84)	2.51 (0.26)	0.526 (0.046)	0.545 (0.049)	0.028 (0.013)	28	53.51.	0.003	sig
	SalE2	323	10	2.43	0.591	0.588	−0.005	45	40.90	0.646	ns
	Sal3	323	9	2.41	0.545	0.585	0.068	36	46.19	0.119	ns
	SalE5	323	5	1.04	0.037	0.037	−0.015	10	0.12	1.000	ns
	SalE6	323	4	2.30	0.517	0.565	0.085	6	5.45	0.488	ns
	SalE7	323	11	2.78	0.628	0.640	0.019	55	26.77	1.000	ns
	SalE8	322	8	4.12	0.727	0.758	0.041	28	42.99	0.035	ns
	SalE11	323	6	2.25	0.526	0.556	0.054	15	9.09	0.873	ns
	SalE14	323	6	2.07	0.502	0.518	0.031	15	8.22	0.915	ns
	Sal23	303	9	2.44	0.521	0.589	0.115	36	85.64	≪0.001	sig
	SST-A6-I	323	4	1.63	0.415	0.385	−0.076	6	4.10	0.663	ns
	SST-A6-II	323	8	2.35	0.570	0.575	0.009	28	12.69	0.994	ns
	SST-C2	323	13	2.81	0.625	0.644	0.029	78	84.04	0.300	ns
	SST-C3	323	4	1.66	0.406	0.397	−0.021	6	7.62	0.267	ns
	SST-E11	323	13	4.90	0.752	0.796	0.055	78	78.86	0.451	ns
WF	All loci	124,9 (0.10)	5.86 (0.53)	2.41 (0.21)	0.523 (0.048)	0.540 (0.048)	0.032 (0.020)	26	26.41	0.441	ns
	SalE2	125	8	2.03	0.480	0.506	0.052	28	19.55	0.880	ns
	Sal3	125	7	2.53	0.632	0.604	−0.046	21	13.38	0.895	ns
	SalE5	125	1	1.00	–	–	–	Monomorphic			
	SalE6	125	5	2.38	0.552	0.580	0.048	10	2.41	0.992	ns
	SalE7	125	7	2.62	0.680	0.618	−0.100	21	18.93	0.589	ns
	SalE8	124	9	4.39	0.742	0.772	0.039	36	37.23	0.412	ns
	SalE11	125	5	2.12	0.480	0.528	0.091	10	7.53	0.675	ns
	SalE14	125	4	2.42	0.592	0.586	−0.010	6	2.98	0.811	ns
	Sal23	125	6	2.50	0.616	0.600	−0.026	15	11.35	0.728	ns
	SST-A6-I	125	6	2.01	0.432	0.503	0.141	15	128.45	≪0.001	sig
	SST-A6-II	125	6	2.29	0.600	0.564	−0.063	15	8.34	0.909	ns
	SST-C2	124	7	3.26	0.605	0.693	0.127	21	9.49	0.985	ns
	SST-C3	125	4	1.67	0.384	0.403	0.047	6	1.58	0.954	ns
	SST-E11	125	7	2.48	0.528	0.596	0.115	21	21.38	0.436	ns

sig, significant; ns, not significant.

Mean population parameters did not significantly differ between the two groups (Table 3). Nonetheless, the PEF population had a significantly larger number of sampled individuals than the WF and a higher number of private alleles (37 in the PEF and 9 in the WF). In particular, the SalE5 locus became monomorphic in the WF subsample (Table 3).

In the second step, we re-ran a population structure analysis separately for each of the two clusters previously identified (PEF and WF groups). We first tested for HWE using GeneAlex and deleted from the analyses all loci not at equilibrium after Holm–Bonferroni correction and with null alleles. In the PEF group, only Sal23 was out of HWE, while in the WF group, SalE5 resulted monomorphic and SST-A6-I not at HWE. We therefore ran population structure without locus Sal23 for the PEF and without loci SalE5 and SST-A6-I for the WF. Neither of the “independent allele frequencies” models, ran in this step for both the PEF and the WF, converged, even increasing the length of *burnin* and MCMCs, respectively to 100,000 and 1,000,000. We therefore considered only the “correlated allele frequencies” models hereafter.

The results of the PEF group were similar in terms of the number of clusters identified using the “admixture” and “no-admixture” models. Both models identified two clusters, according to both the Evanno and Pritchard methods. The “admixture model” showed a distinct cluster corresponding to SP12 with a *Q*_p_ of 67%. All others SPs were assigned to a single cluster, with *Q*_p_s always higher than 69%. The “no-admixture” model supported the presence of 2 clusters (*K* = 2), and the results were consistent with the “admixture” model, identifying SP12 as a single cluster (*Q*_p_ = 94%) and all other SPs pertaining to a second cluster (*Q*_p_ always higher than 85%), except for SP10 which had a *Q*_p_ of 67% (see Fig. 3B for the “no-admixture” model after CLUMPP analysis). Although the mean population genetic parameters did not significantly differ between the two clusters of the PEF (Table 4a), their Global *F*_ST_ was significantly different from zero (13 loci, *F*_ST_ = 0.006, *P *< 0.001). The two clusters showed a different number of private alleles, 33 in cluster 1 and 1 in cluster 2 (SP12). The latter also showed the locus SalE5 as monomorphic (Table 4a).

**Table 4:** Population genetic parameters (with SE in parentheses) for the (a) two clusters of the PEF group and (b) four clusters of the WF group: number of genotyped individuals (*N*), number of different alleles (Na), number of effective alleles (Ne), allelic range (AR), observed (Ho) and expected heterozygosity (He), and fixation index (*F*_IS_). Hardy–Weinberg Equilibrium test: degree of freedom (Df), chi-squared value (Chi-sq), *P*-value (p), significance after Holm–Bonferroni correction (HB sig).

		Population genetic parameters						HWE test			
Cluster	Locus	N	Na	Ne	Ho	He	*F*_IS_	DF	Chi-sq	*P*	HB sig
(a)											
1	All loci	278.9 (0.08)	7.69 (0.91)	2.51 (0.27)	0.525 (0.049)	0.542 (0.053)	0.023 (0.011)	26	34.88	0.114	ns
	SalE2	279	10	2.41	0.577	0.584	0.013	45	49.43	0.301	ns
	Sal3	279	9	2.36	0.538	0.576	0.066	36	46.72	0.109	ns
	SalE5	279	5	1.04	0.043	0.042	−0.017	10	0.13	1.000	ns
	SalE6	279	4	2.31	0.538	0.568	0.053	6	4.88	0.559	ns
	SalE7	279	11	2.86	0.645	0.651	0.009	55	26.16	1.000	ns
	SalE8	278	8	4.00	0.723	0.750	0.036	28	37.93	0.100	ns
	SalE11	279	6	2.28	0.516	0.562	0.081	15	11.44	0.721	ns
	SalE14	279	6	2.09	0.502	0.522	0.038	15	8.36	0.909	ns
	SST-A6-I	279	4	1.61	0.401	0.378	−0.062	6	2.06	0.914	ns
	SST-A6-II	279	7	2.36	0.559	0.577	0.031	21	13.83	0.877	ns
	SST-C2	279	13	2.94	0.631	0.659	0.043	78	81.94	0.358	ns
	SST-C3	279	4	1.64	0.405	0.390	−0.037	6	11.69	0.069	ns
	SST-E11	279	13	4.70	0.749	0.787	0.048	78	75.77	0.550	ns
2	All loci	44	5.23 (0.63)	2.47 (0.33)	0.533 (0.055)	0.522 (0.054)	−0.025 (0.031)	26	22.37	0.668	ns
	SalE2	44	7	2.54	0.682	0.607	−0.123	21	12.30	0.931	ns
	Sal3	44	5	2.71	0.591	0.631	0.064	10	27.40	0.002	ns
	SalE5	44	1	1.00	–	–	–	Monomorphic			
	SalE6	44	4	1.87	0.386	0.465	0.168	6	7.20	0.302	ns
	SalE7	44	6	2.22	0.523	0.550	0.050	15	12.57	0.635	ns
	SalE8	44	6	4.50	0.750	0.778	0.036	15	12.42	0.647	ns
	SalE11	44	4	2.07	0.591	0.517	−0.142	6	6.72	0.347	ns
	SalE14	44	3	1.96	0.500	0.491	−0.019	3	1.27	0.737	ns
	SST-A6-I	44	4	1.75	0.500	0.429	−0.165	6	5.89	0.436	ns
	SST-A6-II	44	7	2.21	0.636	0.547	−0.163	21	6.19	0.999	ns
	SST-C2	44	7	2.12	0.591	0.529	−0.118	21	13.13	0.904	ns
	SST-C3	44	4	1.77	0.409	0.435	0.059	6	1.63	0.951	ns
	SST-E11	44	10	5.33	0.773	0.812	0.049	45	46.25	0.420	ns
(b)											
1	All loci	56	5.83 (0.44)	2.47 (0.17)	0.555 (0.036)	0.574 (0.028)	0.037 (0.030)	24	20.35	0.677	ns
	SalE2	56	8	1.92	0.446	0.479	0.068	28	20.53	0.845	ns
	Sal3	56	7	2.50	0.571	0.601	0.049	21	12.35	0.930	ns
	SalE6	56	5	2.28	0.446	0.561	0.204	10	5.28	0.872	ns
	SalE7	56	7	3.07	0.750	0.674	−0.112	21	16.89	0.718	ns
	SalE8	56	8	3.77	0.750	0.735	−0.021	28	16.19	0.963	ns
	SalE11	56	4	1.94	0.411	0.484	0.151	6	6.47	0.373	ns
	SalE14	56	4	2.14	0.607	0.533	−0.138	6	2.72	0.843	ns
	Sal23	56	5	2.39	0.571	0.582	0.018	10	4.40	0.928	ns
	SST-A6-II	56	5	2.34	0.607	0.573	−0.060	10	4.55	0.919	ns
	SST-C2	56	7	3.11	0.625	0.679	0.079	21	9.02	0.989	ns
	SST-C3	56	4	1.62	0.357	0.383	0.069	6	2.40	0.879	ns
	SST-E11	56	6	2.52	0.518	0.604	0.143	15	14.55	0.484	ns
2	All loci	3	2.83 (0.37)	2.48 (0.31)	0.639 (0.119)	0.505 (0.075)	−0.242 (0.140)	–	–	–	–
	SalE2	3	3	2.57	1.000	0.611	−0.636	3	3.00	0.392	ns
	Sal3	3	3	2.57	1.000	0.611	−0.636	3	3.00	0.392	ns
	SalE6	3	2	1.80	0.000	0.444	1.000	1	3.00	0.083	ns
	SalE7	3	4	3.60	1.000	0.722	−0.385	6	4.50	0.609	ns
	SalE8	3	5	4.50	1.000	0.778	−0.286	10	9.00	0.532	ns
	SalE11	3	1	1.00	–	–	–	Monomorphic			
	SalE14	3	2	1.80	0.667	0.444	−0.500	1	0.75	0.386	ns
	Sal23	3	3	2.57	0.667	0.611	−0.091	3	2.33	0.506	ns
	SST-A6-II	3	2	1.80	0.667	0.444	−0.500	1	0.75	0.386	ns
	SST-C2	3	4	3.00	0.667	0.667	0.000	6	6.33	0.387	ns
	SST-C3	3	1	1.00	–	–	–	Monomorphic			
	SST-E11	3	4	3.60	1.000	0.722	−0.385	6	4.50	0.609	ns
3	All loci	54.83 (0.11)	5.08 (0.43)	2.59 (0.24)	0.587 (0.027)	0.590 (0.025)	0.003 (0.030)	24	24.60	0.428	ns
	SalE2	55	7	1.95	0.436	0.487	0.104	21	23.10	0.339	ns
	Sal3	55	4	2.47	0.655	0.594	−0.101	6	10.72	0.097	ns
	SalE6	55	3	2.48	0.691	0.597	−0.157	3	3.00	0.392	ns
	SalE7	55	5	2.15	0.600	0.536	−0.120	10	3.91	0.951	ns
	SalE8	54	7	4.99	0.759	0.800	0.051	21	26.37	0.193	ns
	SalE11	55	4	2.34	0.582	0.573	−0.016	6	7.59	0.270	ns
	SalE14	55	4	2.56	0.527	0.609	0.135	6	5.36	0.498	ns
	Sal23	55	6	2.55	0.655	0.609	−0.076	15	6.40	0.972	ns
	SST-A6-II	55	5	2.25	0.564	0.557	−0.013	10	2.21	0.994	ns
	SST-C2	54	7	3.24	0.593	0.691	0.142	21	9.77	0.982	ns
	SST-C3	55	3	1.93	0.491	0.482	−0.017	3	0.25	0.969	ns
	SST-E11	55	6	2.21	0.491	0.548	0.104	15	15.16	0.440	ns
4	All loci	11	3.75 (0.18)	2.36 (0.14)	0.591 (0.054)	0.549 (0.044)	−0.074 (0.047)	88	62.32	0.983	ns
	SalE2	11	4	2.55	0.727	0.607	−0.197	6	3.97	0.680	ns
	Sal3	11	4	2.81	0.727	0.645	−0.128	6	2.58	0.859	ns
	SalE6	11	3	2.28	0.545	0.562	0.029	3	1.83	0.608	ns
	SalE7	11	4	2.55	0.636	0.607	−0.048	6	3.20	0.783	ns
	SalE8	11	4	2.44	0.545	0.591	0.077	6	11.55	0.073	ns
	SalE11	11	4	2.30	0.455	0.566	0.197	6	3.28	0.772	ns
	SalE14	11	4	2.92	0.818	0.657	−0.245	6	4.27	0.640	ns
	Sal23	11	4	2.20	0.636	0.545	−0.167	6	1.67	0.947	ns
	SST-A6-II	11	4	2.18	0.727	0.541	−0.344	6	3.59	0.732	ns
	SST-C2	11	4	2.72	0.545	0.632	0.137	6	6.93	0.327	ns
	SST-C3	11	2	1.10	0.091	0.087	−0.048	1	0.02	0.875	ns
	SST-E11	11	4	2.22	0.636	0.550	−0.158	6	3.85	0.696	ns

sig, significant; ns, not significant.

The analysis of the WF group produced different results using the “admixture” and “no-admixture” models, because the second one identified a more subtle structure. In the “admixture” model, the Evanno method allowed us to identify two clusters, with *Q*_p_s always higher than 73%. According to the Pritchard method, three clusters could be identified, although in this case, *Q*_p_s were never higher than 63% and it was not possible to assign each SP to a defined population of origin. Conversely, the results of the “no-admixture” model suggested two clusters using the Evanno method and three to four clusters using the Pritchard one. In this model, *Q*_p_s were generally rather higher than the previous model allowing us to ascertain a stronger genetic structure. Assuming *K* = 2, *Q*_p_s were always higher than 86%, assigning SP5 and SP6 to cluster 1 and SP7 and SP8 to cluster 2. Considering *K* = 3 or *K* = 4, the general pattern of population structure did not change (*Q*_p_s > 84% [*K* = 3] and *Q*_p_s > 69% [*K* = 4] pertaining to the clusters 1 or 2). Nonetheless, the *Q*_p_s pertaining to cluster three or four were relatively low for all SPs. These two clusters were represented by two groups of genetically distinct individuals, each of which collected in a unique sampling location. The group assigned to cluster 3 was split from SP5 (*Q*_i_s > 93% with *K* = 3 and *Q*_i_s > 99% with *K* = 4), and the group assigned to cluster 4 was split from SP7 (*Q*_i_s > 66%) (see Fig. 3C for the “no-admixture” model after CLUMPP analysis). The mean population genetic parameters were not significantly different among the four cluster of the WF group, except for the number of different alleles (Na; Table 4b). In cluster 2, locus SalE11 and SST-C3 resulted monomorphic (Table 4b). Global *F*_ST_ for the WF group was 0.017 (12 loci: *P *= 0.058). Pairwise *F*_ST_ among the four clusters of the WF group ranged between 0.007 and 0.083, but none of them were significantly different from zero after sequential Bonferroni correction (Table 5).

**Table 5:** Pairwise *F*_ST_ among the four clusters of the Western foothill lowland (WF) group below the diagonal. Probability based on 9999 permutations is shown above the diagonal. None of these values were significant after a Holm–Bonferroni correction.

Cluster	1	2	3	4
1	–	0.323	0.043	0.045
2	0.052	–	0.181	0.048
3	0.007	0.059	–	0.029
4	0.022	0.083	0.022	–

## Discussion

This research allowed us to detect a hierarchical population structure of the Fire Salamander in a wide area of the species range in Northern Italy. The 28 sampled populations were distributed in the Prealpine area, where forest cover is more continuous, and in the foothill lowland, where forests are more fragmented (see Fig. 1). Some SPs from the Western foothill lowland appeared genetically distant from the others, according to pairwise *F*_ST_ (Table 2). This result was confirmed by genetic structure analyses which highlighted how these populations were strongly structured. We identified two groups, one represented by the Western Foothill populations (WF) and another one by all other populations laying in the Prealpine and Eastern Foothill areas (PEF). This was confirmed by the overall high probability of assignment of individuals to the two groups which resulted genetically distant as shown by significant pairwise F_ST_. Even if locus SalE5 became monomorphic in the WF group, the number of effective alleles (Ne) and the observed heterozygosity (Ho) were very similar between PEF and WF, suggesting that currently there was not a loss of genetic diversity on the whole.

After the identification of these two genetically separated groups, the use of a multistep approach allowed us to identify substructured populations.

In the PEF groups, two more clusters were identified: A first one included all populations except SP12, which formed a second cluster whose separation was confirmed by both population structure analysis and pairwise *F*_ST_. SP12 cluster is located in a foothill forest separated from Prealpine areas by both wide conurbation and a main road. In addition, this area was also found to be isolated by the lack of a stream network connection that may support the dispersal process and seems to be important in connecting other Eastern foothill populations to the Prealps.

**Figure 4:**
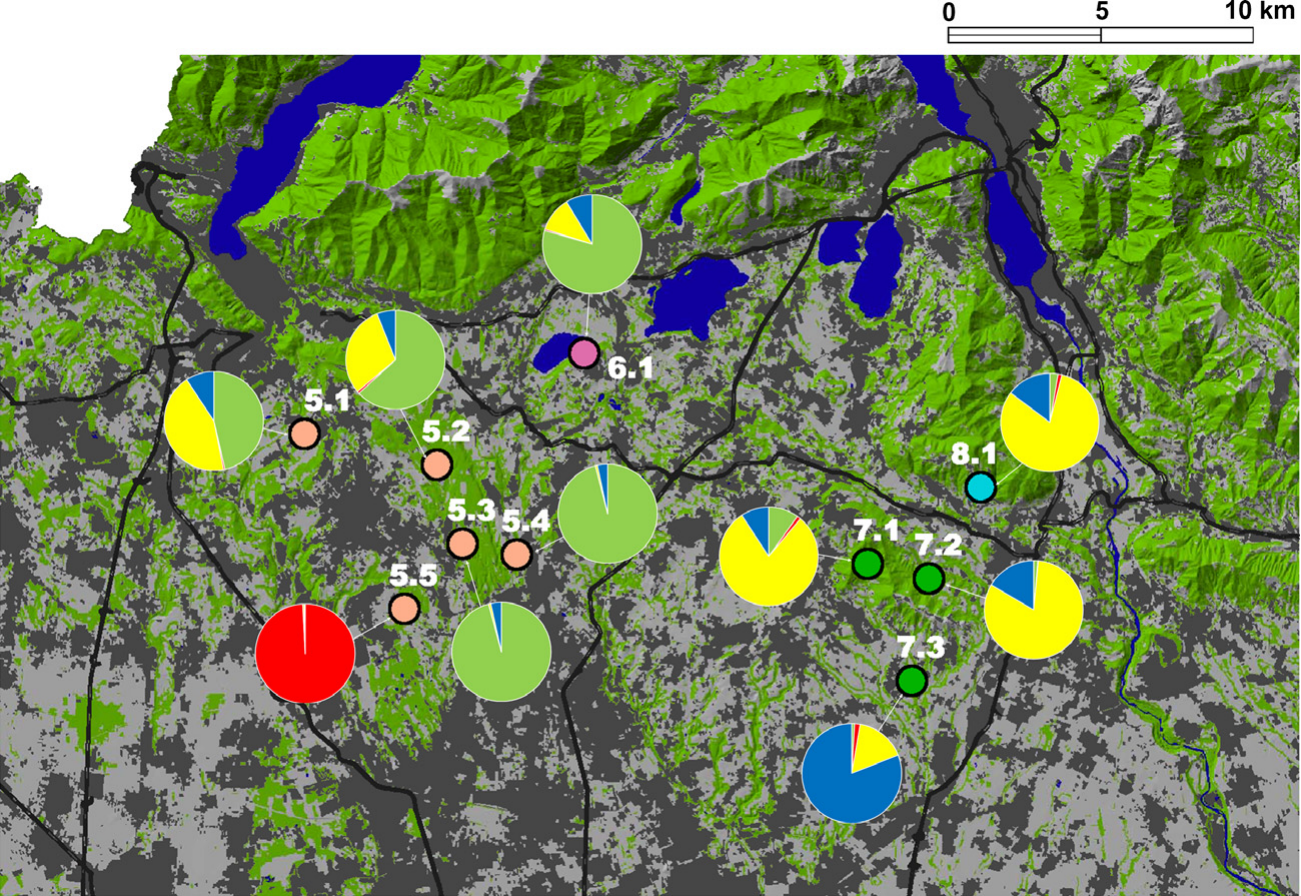
Population structure of the Western foothill lowland (WF) group. Numbered dots indicate sampling locations, grouped according to their sampling population (dot colors). Pie charts represent the sampling location probability of assignment to the four clusters identified by running STRUCTURE. Cluster 1: green, cluster 2: red, cluster 3: yellow, and cluster 4: blue. These colors correspond to those in Fig. 3C with *K* = 4. In the background main land uses: forests in green, urban areas in dark–gray, farmland, and other open habitats in gray, lakes, wetlands, and rivers in blue. Black lines represent main roads.

The WF group could be divided from two to four groups adopting the Evanno or Pritchard method. Considering *K* = 2, the group is divided into a Western (SP5 and SP6) and Eastern (SP7 and SP8) cluster, divided by large conurbations and a south–north main road (Fig. 4). In addition, according to the *K* = 4 result, we observed another subdivision occurring in the two previous clusters (cluster 1: SP5 and SP6; cluster 3: SP7 and SP8), identifying two new clusters corresponding to two sampling locations, sampling location 5.5 (cluster 2), and sampling location 7.3 (cluster 4). Although these two new clusters are very close (with a distance of about three kilometers) to the other sampling locations of the same SP, they seem to represent separated demes. Cluster 2 appeared to be almost completely isolated by unsuitable areas for the Fire Salamander, largely represented by urban areas without stream network connections, while cluster 4 is isolated mainly by agricultural areas but with a stream connection, which could make the isolation less sharp (Fig. 4). Nevertheless, none of the pairwise *F*_ST_ tests for the WF group resulted significant after Holm–Bonferroni correction. This result may be conditioned by the small sample size of clusters 2 and 4, each represented by a single sampling location, with respect to the SPs they were split from.

On the whole, the identified population structure may be explained by the hypothesis of a relatively short-term fragmentation in the WF group: In this case, we cannot currently detect a significant loss of alleles, which could arise in the long term due to an inadequate gene flow. Conversely, it is likely that the PEF group (except SP12) will maintain a high number of alleles in the long term, because it is represented by a large and mainly continuous population (Fig. 3B).

Despite the fragmentation process currently only led to an evident population structure without significant differences of genetic parameters among identified clusters, in the medium and long term, it is expected it could even produce a loss of genetic diversity (e.g., Dixo et al. 2009). Indeed, the observed strong genetic population structure within the WF group is particularly worrying, because this will probably lead to a reduction in the capacity of adaptation of these populations, resulting in an increase in their probability of extinction, in relatively short time (Young et al. 1996; Reed and Frankham 2003; Arens et al. 2007). In addition, these populations will face other important threats such as further habitat loss and degradation caused by infrastructural, as well as commercial/residential development projects in their area, which has one of the highest rate of urbanization in Italy. The same fate of the WF is expected for SP12, split from the PEF group, although its isolation seems to be less strong than the one occurring between the WF and the PEF groups and within the WF group.

From a methodological point of view, the multistep approach we adopted for the genetic population structure analysis at large spatial scale allowed us to identify different cases of fragmentation in the overall population, likely corresponding to a different degree of separation, or even isolation, of populations (see sampling location 5.5, i.e., cluster 2 of WF group). The effectiveness of this approach was proved by the use of the species here investigated, the Fire Salamander, an amphibian with rather strict ecological requirements (Di Cerbo and Razzetti 2004; Schmidt et al. 2005). Nonetheless, the genetic results suggest that at wide scale, most of the salamander populations are rather continuous or large enough to not suffer the typical problem of small population; they also highlight how locally the species may suffer habitat fragmentation. This is confirmed by the presence of strong structured populations, with a generalized genetic depletion, evidenced by the presence of some monomorphic loci and many private alleles.

In conclusion, the multistep approach can be useful to detect substructured populations at local scale which cannot be observed analyzing wide-scale data in one-step only. Thus, the screening of genetic population structure from a regional to local scale can be considered effective in detecting local populations of conservation concern which resulted imperilled by threats acting locally and also for a preliminary assessment of the ecological connectivity in fragmented landscapes.

## Data Accessibility

Sampled individuals, sampling locations, and microsatellite genotypes: Dryad
